# Co-Sleeping between Adolescents and Their Pets May Not Impact Sleep Quality

**DOI:** 10.3390/clockssleep3010001

**Published:** 2021-01-04

**Authors:** Jessica Rosano, Tiffani Howell, Russell Conduit, Pauleen Bennett

**Affiliations:** 1 Anthrozoology Research Group, School of Psychology and Public Health, La Trobe University, Bendigo, VIC 3552, Australia; jessicarosano550@gmail.com (J.R.); pauleen.bennett@latrobe.edu.au (P.B.); 2 School of Health and Biomedical Sciences, RMIT University, Melbourne, VIC 3083, Australia; russell.conduit@rmit.edu.au

**Keywords:** Pittsburgh Sleep Quality Index (PSQI), global sleep score, dog, cat, actigraphy, human–animal relationships

## Abstract

Pet–owner co-sleeping is increasingly common in some parts of the world. Adult owners often subjectively report benefits of co-sleeping with pets, although objective actigraphy reports conversely indicate sleep disruptions due to the pet. Because limited research is available regarding pet–owner co-sleeping in non-adult samples, the aim of this two-part study was to explore whether co-sleeping improves sleep quality in adolescents, an age group in which poor sleep patterns are well documented. In Study One, an online survey with 265 pet-owning 13-to-17-year-old participants found that over 78% co-slept with their pet. Average sleep quality scores for co-sleepers and non-co-sleepers indicated generally poor sleep, with no differences in sleep quality depending on age, gender, or co-sleeping status. Study Two consisted of two preliminary case studies, using actigraphy on dog–adolescent co-sleepers. In both cases, high sleep concordance was observed, but owners again experienced generally poor sleep quality. Future actigraphy research is needed, including larger sample sizes and a control group of non-co-sleepers, to validate the preliminary findings from this study, but our limited evidence suggests that co-sleeping with a pet may not impact sleep quality in adolescents.

## 1. Introduction

Pets are an important part of many people’s lives, and many owners allow their pets to ‘co-sleep’ alongside them. Co-sleeping usually refers to human sexual partners or to children who share a bed/bedroom with their caregiver; however, in Western societies co-sleeping arrangements have become common among pet owners [1]. It has been suggested that approximately 50% of dog owners and 60% of cat owners engage in co-sleeping arrangements with their pets [1]. One reason for this may be the sense of security it brings. Sleeping is a vulnerable process and a sense of safety can assist in reducing the psychological arousal that can interfere with sleep onset and quality [2]. Research shows that co-sleeping with a pet encourages these feelings, which may have a positive influence on sleep quality levels [3,4].

Despite the popularity of pets, the literature investigating the impacts of human–animal co-sleeping is limited, with just a few published studies to date. An Australian online survey by Smith et al. [3] explored differences in the relationship between sleep and wellness between adult pet owners and non-owners, and found that pet owners and non-owners were equally likely to wake during the night. Another study asked co-sleeping pet owners whether there were negative impacts from co-sleeping, and about 40% of participants each perceived no ill effects or a beneficial effect, compared to only 20% who reported finding pets disruptive [5]. A qualitative study found that, among seven adults with chronic pain, co-sleeping with their pets could have a beneficial impact on sleep quality, despite typical recommendations that people experiencing chronic pain not co-sleep with their pet [6].

An actigraphy study found that sleep efficiency (i.e., the ratio of total time spent asleep compared to the time spent in bed) was not greatly diminished by co-sleeping with one animal, but having the pet merely in the room promoted better sleep efficiency than if they were on the bed [7]. Two other actigraphy studies found that owner–dog co-sleeping led to mild reductions in sleep quality in owners, due to animal movement [8,9]. Taken together, findings from surveys and actigraphy studies suggest that co-sleeping status may affect sleep quality for some, but not all individuals. It is not immediately clear why people are affected differently, but sleep–wake concordance could be a factor influencing sleep quality among people who co-sleep with animals.

Couples who share a bed often experience similar sleep and wake times, whilst also demonstrating similar movement [10]. Previous research has found that sleeping concordantly among adult couples is associated with higher marital satisfaction [11] and reduced risk of cardiovascular disease [12], but not sleep quality [11,12]. Even though sleep quality was not related to sleep concordance in studies of human dyads, since animal movement was related to human sleep disruptions in pet–owner actigraphy research [8,9], this could cause sleep deficits in co-sleeping pet owners.

It is likely that adolescents are frequent human–animal co-sleepers, as pets are more likely to be found in households with teenagers [13,14]. Studies have investigated the impact of pets on child and adolescent development, with a systematic review finding social, emotional, and cognitive benefits of pet ownership in adolescents [15]. However, there are no studies exploring pet co-sleeping in teenagers. Typically, adolescents require between eight and 10 h of sleep per night [16], with most only sleeping approximately seven hours [17]. During adolescence, sleep quality has been shown to be impacted by the multiple demands placed on teenagers, such as balancing school, work, and hobbies [18], as well as possible dysregulation of circadian rhythms due to hormonal changes [19] or associated emotional or affective challenges. It is unclear whether co-sleeping with pets has an impact on sleep quality in adolescents. Co-sleeping could conceivably improve sleep by reducing anxiety and promoting a sense of security, because there is evidence of a weak within-person bidirectional relationship between anxiety and sleep quality [20], but there is mixed evidence for the impact of pets on anxiety [15]. Alternatively, it could disrupt sleep due to poor sleep concordance.

The aim of the current study was to explore the impacts of human–animal co-sleeping on overall sleep quality in adolescents. To achieve this aim, one survey study and one actigraphy study were employed. The survey study investigated the impact of co-sleeping on self-reported sleep quality in an adolescent population, to understand whether co-sleepers would report better or worse sleep quality than non-co-sleepers. The second study, the exploratory study, used actigraphy to determine objective sleep parameters (i.e., sleep onset and wake times) by measuring activity patterns in two dog–owner dyads. In this preliminary study, dog–owner sleep–wake concordance was measured in two adolescent females and their pet dogs, along with self-reported sleep quality.

## 2. Results

### 2.1. Study One—Survey

According to the results of our online survey of adolescents, most respondents (*n* = 208, 78%) co-slept with their pet, whereas 57 (22%) did not. Among co-sleepers, 196 participants (74% of the total sample) reported that their pet slept on their bed, while the remaining 12 respondents (5% of the total sample) indicated that their pet slept in their room but not on the bed. Used as a measure of sleep quality, global sleep scores on the Pittsburgh Sleep Quality Index (PSQI) ranged from 1 to 18 out of a total hypothetical range of 0–21. The PSQI measures seven factors impacting sleep quality (e.g., latency to sleep and sleep disturbances), as well as providing a global sleep-quality score. In accordance with Buysse et al. [21], a global score greater than 5 was taken to indicate a severe sleep deficit. Most survey respondents (*n* = 172, 65%) scored 6 or higher. Descriptive results for individual PSQI components and the global sleep score, grouped by co-sleeping status, are presented in Table 1. Data from one non-co-sleeper were excluded due to missing data. Medians are presented rather than mean/standard deviation because all data were non-normally distributed.

The median global sleep scores for all three groups indicated a poor quality of sleep. When the three groups were compared on component and global sleep scores, independent-samples Kruskal–Wallis tests indicated no difference between groups on any domains or on global sleep quality (see Table 1). Similarly, no difference was observed when comparing age (*H* = 4.94, *df* = 2, and *p* = 0.085) or gender (*H* = 1.30, *df* = 2, and *p* = 0.523) on co-sleeping status. As a secondary analysis, when all co-sleepers (i.e., room and bed) were combined into a single group and compared with non-co-sleepers, a Mann–Whitney *U*-test showed no differences between groups on age (*U* = 5166.50, *z* = −1.58, *p* = 0.114) or gender (*U* = 5770.00, *z* = −0.525, and *p* = 0.600). There was also no effect of co-sleeping status on sleep quality variables, as shown in Table 2.

### 2.2. Study 2—Actigraphy Pilot

#### 2.2.1. Self-Reported Owner–Dog Co-Sleeping Status and Sleep Quality

Both participants completed a paper version of the survey from Study One, providing information about co-sleeping with their dog. Case Study 1 reported that her small dog slept on her bed for 8 to 10 h per night, seven nights per week. Case Study 2 indicated that her large dog slept in her room seven nights per week, for 8 to 10 h per night, but only slept on the bed four nights per week, for 1 to 3 h per night. In both cases, the received signal strength indicators (RSSI) from the actigraphy software recorded between 0.70 and 0.81, indicating that the owner and dog were in close proximity.

In the paper survey, they also provided subjective reports of their sleep quality. PSQI scores >5 indicated severe sleep deficits in both cases; Case Study 1 one obtained a global score of 8, and Case Study 2 obtained a score of 5 (see Table 3 for PSQI component scores for each case).

#### 2.2.2. Sleep–Wake Concordance

In order to generate concordance values, sleep reports were created based on activity data received via actigraphy devices over two weeks. Each report provided sleep parameters, including total time in bed, total sleep time, and wake after sleep onset. Full actigraphy reports for both case studies are available in the Supplementary Materials. Sleep reports for each dyad were used to compute concordance from sleep onset in the humans, for four combinations: the human was sleeping, whilst the dog was awake; the human was awake, whilst the dog was sleeping; the human and dog were sleeping at the same time; and the human and dog were awake at the same time (see Table 4).

Case Study 1 had a sleep concordance percentage of 83.4%, indicating consistent sleep. Case Study 2 had a sleep concordance of 75.7%. It was rare for the owner and dog to be awake at the same time in either case. It was more common for the dog to be awake while the owner was asleep. There was no clear association between sleep concordance and self-reported sleep quality for these two participants. Case Study 1 reported worse sleep quality but had a higher percentage of owner–dog sleep concordance than Case Study 2, who indicated better sleep but with lower sleep concordance.

## 3. Discussion

The present study aimed to investigate the impact of human–animal co-sleeping on sleep quality in an adolescent population. This was achieved through two studies, an online survey examining cross-sectional data from a sample of adolescent pet-owners, and two small case studies, in which co-sleeping behaviour was tracked for two weeks. The only other known studies using actigraphy to explore owner–pet co-sleeping are by Hoffman et al. [9], Patel et al. [7], and Smith et al. [8], all of which applied actigraphy to adult owners and their dogs. This study differed from previous research, as it focused on a younger demographic.

In Study One, we investigated whether adolescents who co-slept with their pets would differ in sleep quality than those who did not. There were no differences between adolescents who reported co-sleeping with their pet and those who did not. Global sleep scores indicated poor sleep among non-co-sleepers and co-sleepers alike. Study Two measured activity patterns to determine sleep parameters in two case studies. Sleep–wake concordance was used to provide objective data on whether there was a positive relationship between concordance between pet and owners, and the owner’s sleep quality. In case study one, the owner had severe sleep difficulties despite high pet–owner sleep–wake concordance. In Case Study 2, the global score indicated better quality sleep than Case Study 1, but sleep–wake concordance was slightly lower than for Case Study 1.

A majority (65%) of adolescents in the study had global sleep scores that indicated severe sleep deficits. This is consistent with previous research, which has shown that insufficient or poor sleep is common for adolescents [18,22,23], as well as in children as young as eight years old [24]. The PSQI global score for adolescents in the current study was similar to that reported in previous studies [25,26,27]. Based on previous findings, sleep difficulties in the current sample may reflect adolescents’ inability to effectively regulate sleep habits.

Co-sleeping with pets has been identified as a contributor to sleep disruptions when measured using actigraphy [8,9]. This has been supported in validated survey measures of sleep quality [8], but not subjective self-reports (i.e., sleep diary [9]). Our findings demonstrate that sleep quality in adolescents who reported co-sleeping with their pets was no better and no worse than sleep quality in those who did not co-sleep. Consequently, disturbances were not a cause of poor sleep quality in the current sample. This outcome may be explained by the sleep–wake concordance observed in Study Two, as a high degree of sleep concordance suggests that owner sleep is not routinely disrupted by their pets.

Sleep concordance in human couples has been shown to range between 53% and 88% [11,12]. Concordance for both cases in Study Two fell within this range. The proportion of time the owner spent awake whilst the dog was asleep was very low in both cases, as was the percentage of time when both dyad members were awake. These low values indicate minimal disruptions between dyads. If pet-owners who co-sleep with their pets experience few disruptions, then the dyad may have similar sleep experiences as non-co-sleepers, which may further explain the lack of differences between groups in Study One. This cannot be concluded due to the limited sample, but these findings merit further investigation.

The current study has endeavoured to begin to address the lack of empirical research regarding the influence of human–animal co-sleeping on sleep quality in an adolescent population. This study shifted the focus away from adult populations who co-sleep with significant others or their child, towards a younger demographic who do, or do not, co-sleep with pets. In this population, characterised by generally poor sleep quality, we found no evidence that co-sleeping with a pet improves or impairs sleep quality. We recommend that further research consider potential moderating factors, such as school schedules, jobs, and parental behaviours (e.g., curfews and bedtimes) [18], which were not addressed in the current study’s design. It remains possible that, for a subsample of adolescents, sleeping with pets may have strong negative or positive consequences, beyond those that could be revealed by using our methodology. A particularly active pet, for example, may disrupt sleep. Conversely, for particularly anxious adolescents, co-sleeping with a pet may dissipate arousal and improve sleep quality. Furthermore, with our current dataset, we were unable to compare adolescents who co-sleep with cats or dogs. Future research should investigate whether pet species impacts sleep quality.

In Study One, there was a lack of gender and age diversity amongst participants, despite the sufficient sample size. Furthermore, we relied on a convenience sample of participants, so it is not representative of all teenagers. Nearly all adolescents identified as females, and the median age was 16 years, limiting the generalisability of the results to other adolescent populations. Study Two findings yielded interesting results, but they should be considered against multiple limitations. The study was designed to be preliminary, but the small sample size means that wider conclusions cannot be drawn based on these data. Furthermore, despite being validated on dogs [28], there were no identified algorithms in the actigraphy software for scoring dogs’ sleep. Thus, parameters were calculated by using algorithms designed to score children’s sleep. This means it may not transfer well to dogs, due to their being alert but physically inactive when resting [29]. Indeed, actigraphy determines sleep based on inactivity, rather than brain activity, so it is unable to differentiate between rest and sleep. There is also preliminary evidence in humans that actigraphy can vary depending on which limb is used to wear the device, with lower limbs recording different levels of activity than upper limbs [30]. An activity diary was used to collect sleep/wake information to validate human sleep parameters, but no such diary was kept about the dog’s sleep/wake patterns. This means some of the ‘sleep’ periods in the dogs, as indicated by the actigraphy software, may have been periods of wakeful rest. Future research should also incorporate the use of a sleep/activity diary for pets, whereby owners briefly indicate times they notice their dog asleep/awake to validate sleep and wake times indicated by actigraphy devices. Finally, using a larger sample and an experimental and control group (e.g., pet and owner co-sleepers vs. non-co-sleepers) could help consolidate these findings.

## 4. Materials and Methods

### 4.1. Study One—Survey

Study One used an online questionnaire to measure whether sleep quality differed by co-sleeping status among adolescents. 

#### 4.1.1. Participants

A total of 315 individuals responded to an online survey via the Qualtrics survey platform. Fifty cases were excluded from the study due to incomplete responses or failing to meet the inclusion criteria. Therefore, 265 individuals remained, most of whom were female (*n* = 230, 87%) and aged between 13 and 17 years (M = 15.89, SD = 1.34, and median = 16). Of these, 217 participants (82%) owned a dog, 119 (45%) owned a pet cat, and 32 (12%) had a pet categorised as ‘other’. Based on free-text responses, this category included birds, chickens, horses, fish, rabbits, guinea pigs, lizards, and snakes. This sample size was adequate, according to an a priori power analysis, which determined that a minimum sample size of 85 would be required to observe a medium effect.

#### 4.1.2. Materials

A survey was created to measure sleep quality in adolescents. It employed a validated measure of sleep quality, as well as demographics measures which were created specifically for this study by the research team. In the interest of time taken to complete the survey, only one measure of sleep quality was employed.

Demographics Questionnaire. A demographics questionnaire was created to address the following: age, gender, if the respondent had a pet, if the pet slept on the bed/in the room (including how often), and bed size. The questionnaire also collected information regarding how many people lived in the household (see Supplementary Materials).

Pittsburgh Sleep Quality Index. The Pittsburgh Sleep Quality Index (PSQI) measures sleep quality over the previous month, across several domains: subjective sleep quality, sleep latency, sleep duration, habitual sleep efficiency, sleep disturbances, use of sleep medication, and daytime dysfunction [21]. Scores are given for each component and are then summed, to yield a global score. This score ranges between 0 and 21. Scores greater than 5 indicate severe sleep difficulties in at least two areas, or moderate difficulties in more than three [21].

The PSQI was originally developed for adult populations, though its use has been validated with younger people [31]. Those authors modified item 8, which originally read as, “During the past month, how often have you had trouble staying awake while driving, eating meals, or engaging in social activity?” by replacing ‘driving’ with ‘studying’. This version demonstrated moderate validity (Cronbach’s α= 0.72) and was, therefore, used in the current study. A later study further validated the PSQI in teenagers [32] and found a similar internal consistency (Cronbach’s α= 0.73).

#### 4.1.3. Procedure

Participants accessed the survey via a link to the Qualtrics survey platform, advertised on social media. The main source of recruitment was via pet- and adolescent-related groups on Facebook. The recruitment advertisement, which contained a link to the survey, asked adolescents with a cat or dog whether their pet influences how well they sleep. It then indicated the approximate time taken to complete the survey (i.e., 20 min), and the inclusion criteria. Inclusion criteria consisted of owning a pet, being between 13 and 17 years of age, and being able to understand English. This resulted in a convenience sample of cat- or dog-owning teenagers. Participants gave informed consent by checking a box and were then able to complete the survey. Upon completion, participants were presented with a description of a follow-up study (i.e., Study Two). If they were interested in participating, they provided their contact details via a separate link, in order to protect the anonymity of their survey responses. All participants were able to enter a prize draw to win one of four $50 Visa prepaid gift vouchers, also via a separate link. The survey took approximately 10 min to complete, and participants were able to withdraw from the study by exiting the website.

#### 4.1.4. Data Analysis

All data were analysed by using IBM SPSS Statistics version 25 (Armonk, New York, USA). Co-sleeping was calculated based on whether the pet slept in the owner’s room, on the owner’s bed, or neither. If the owners reported that the pet slept in their room or on their bed at least one night during a typical week, they were considered co-sleepers, regardless of the number of hours that the pet was likely to remain in the owner’s room or on the bed. According to the results of Kolmogorov–Smirnov tests, all variables included in analysis were non-normally distributed. Therefore, non-parametric statistics were used. Kruskal–Wallis tests were used to determine if co-sleeping status (i.e., bed, room, or neither) differed by age or gender. The same test was used to measure differences in sleep quality based on co-sleeping status. As a secondary analysis, an independent samples Mann–Whitney U-test was used to compare co-sleepers as a binary variable (i.e., yes or no, rather than separating bed or room co-sleepers) on age, gender, and sleep quality.

### 4.2. Study Two—Actigraphy Pilot

Study Two used actigraphy to measure sleep–wake concordance among two adolescents who co-sleep with their pet dog.

#### 4.2.1. Participants and Recruitment

Individuals who expressed interest via the Study One survey were emailed a description of the follow-up study. Three dog–owner dyads volunteered, but one withdrew from the study because her dog did not tolerate wearing the device around his collar. Therefore, two dyads formed the basis of two case studies.

Participants were required to be between 13 and 17 years of age, able to communicate in English, own a pet dog, co-sleep with it at least some of the time, and reside in metropolitan Melbourne or Geelong, Victoria, Australia. In order to participate in the study, potential participants and a parent/guardian were required to provide written consent. Both participants were female, aged 15 and 17.

#### 4.2.2. Materials

Survey. Participants were given the same questionnaire that they completed in Study One, though it was replicated in paper form. For this study, responses were identifiable.

Sleep/Activity Diary. A sleep and activity diary was used over two weeks. The diary was based on the two week sleep diary published by the American Academy of Sleep Medicine (available from http://yoursleep.aasmnet.org/pdf/sleepdiary.pdf, modified slightly, to add one column for participants to note any times that they were not wearing the actigraphy device (e.g., during a shower). Participants recorded the time they turned the lights off and attempted to sleep, the times that they were asleep, whether spontaneous wakening occurred during the night, and the time they got out of bed the following morning.

Actigraphy. Adolescents wore a wristwatch-like actigraphy device (wGT3-BT monitor; ActiGraph Pty Ltd., Pensacola, FL, USA) on their non-dominant wrist for two weeks. Dogs wore the device around their collar, positioned at the back of their neck. It was secured with cable ties as described in a validation study [28], which concluded that ActiGraph monitors are acceptable for use in dogs. The wGT3-BT model has been demonstrated to be over 90% reliable for seven days of wear, with dog owners reporting the device was well tolerated by their pet [28].

The wGT3-BT monitor recorded continuous physical activity (i.e., movement), which was used to infer total sleep time, sleep onset latency, sleep efficiency, and wake after sleep onset. Long periods of inactivity were recorded as sleep. For the owner, this device was used in conjunction with the sleep/activity diary, in order to validate sleep parameters, as inactivity due to resting can be incorrectly interpreted as a sleep period [8]. Proximity between owner and dog was also recorded, with a received signal strength indicator (RSSI) of at least 0.70 indicating close proximity.

#### 4.2.3. Procedure

Participants who expressed interest in the follow-up study via Study One were emailed a brief description of what was involved. They were also given the opportunity to ask questions. If interested, the potential participant, their parent/guardian and investigator met at the adolescent’s house, to discuss the study. The parent/guardian and adolescent were shown how to use the Actiwatch, with an explanation of how it should be secured to their dog’s collar (i.e., placed on the back of the collar, secured using cable ties). Once the participant and parent/guardian gave written consent, the adolescent completed the paper version of the survey and fitted the Actiwatch to her dog’s collar, with the help of the investigator.

Participants and their dogs wore the device at all times for two weeks, apart from when they were in water. Participants completed the sleep-and-activity diary in order to indicate when they were asleep, awake, or when the Actiwatch was not worn. Participants were not asked to collect this information for their dog. At completion, devices were collected from the participant’s house. They were given a $50 Visa prepaid card and gift basket for their dog, as compensation for their time.

#### 4.2.4. Data Analysis

Actigraphy devices were prepared for data collection and proximity tagging, using ActiLife 6.10 software (ActiGraph Pty Ltd., Pensacola, FL, USA). Data were extracted by using the Cole–Kripke algorithm for human sleep reports [33]. In order to extract data for dog sleep reports, the Sadeh algorithm was used [34]. This is typically used to score children’s sleep, but was used in this study because ActiLife 6.10 does not have a specific algorithm for generating dogs’ sleep parameters. The Sadeh algorithm was deemed appropriate because children and dogs share unpredictable sleep patterns [35].

ActiLife software automatically determined sleep onset and wake times. These times were compared to what was indicated on the sleep and activity diary. If parameters generated by the program were within 30 min of what the participant reported, their information was not changed. If times exceeded 30-min differences, they were altered in accordance with what the participant indicated on the diary. This was because the ActiLife software cannot differentiate between rest and sleep, and previous research has found discrepancies between sleep diary entries and actigraphy measures in adolescents [36].

Sleep reports for each dyad were generated and used to compute concordance for four combinations: the human was sleeping, whilst the dog was awake; the human was awake, whilst the dog was sleeping; the human and dog were awake at the same time; and the human and dog were sleeping at the same time. Sleep concordance was analysed via an Excel worksheet. Wake and sleep times between human and dog were matched at 1-min intervals, coded, and calculated by generating percentages of sleep concordance.

## 5. Conclusions

Understanding the role of co-sleeping in adolescents is important because there are high rates of poor sleep quality in this demographic, and poor sleep quality can seriously impact functioning at a time when optimal functioning is imperative. The project used two studies with the same overall aim, to examine the impact of pet–owner co-sleeping on sleep quality among adolescents. Study One demonstrated that adolescents who co-slept with their pets did not differ in sleep quality compared to those who did not. Thus, co-sleeping did not either improve or impair sleep quality. Study Two highlighted that sleeping concordantly with a dog did not make a difference as to how well the individual slept. Given the limited sample size and the use of an algorithm designed for child sleep in measuring dog activity, these findings should be interpreted with caution. At present, it appears that co-sleeping is not a ready antidote for poor sleep quality in adolescents, nor does it worsen sleep quality. Future research is required to determine if specific subsamples do show significant effects.

## Figures and Tables

**Table 1:** Median (Md), minimum, and maximum scores on the Pittsburgh Sleep Quality Index (PSQI) components and global score for 56 non-co-sleepers, 12 pet–owner co-sleepers (room), and 192 co-sleepers (bed) who completed the online survey. A global score of >5 indicates severe difficulties in at least two domains, or moderate difficulties in more than three. Kruskal–Wallis test *H*-scores and *p*-values are also presented. Degree of freedom for all tests was 2. All results were non-significant.

PSQI Factor	Non-Co-Sleeper			Co-Sleeper (Room)			Co-Sleeper (Bed)			*H*-Score	*p*-Value
	Md	Min	Max	Md	Min	Max	Md	Min	Max		
Subjective sleep quality	1	0	3	1	0	2	1	0	3	0.73	0.695
Latency to sleep	1	0	3	1	0	3	2	0	3	3.05	0.218
Sleep duration	0	0	3	0	0	1	0	0	3	0.98	0.613
Habitual sleep efficiency	0	0	3	0	0	1	0	0	3	0.15	0.928
Sleep disturbances	1	1	3	2	1	2	1	0	3	1.76	0.414
Use of medication	0	0	3	0	0	2	0	0	3	0.21	0.903
Daytime dysfunction	2	0	3	1.5	1	3	2	0	3	0.60	0.740
**Global sleep score**	**6**	**2**	**18**	**6**	**3**	**12**	**7**	**1**	**14**	**1.78**	**0.410**

**Table 2:** Median (Md), minimum, and maximum scores on the PSQI components and global score 56. non-co-sleepers and 208 co-sleepers (room or bed) who completed the online survey. A global score of >5 indicates severe difficulties in at least two domains, or moderate difficulties in more than three. Mann–Whitney *U*-test scores, *z*-scores, and *p*-values are also presented. All results were non-significant.

PSQI Factor	Non-Co-Sleeper			Co-Sleeper (Room or Bed)			*U*-Score	*z*-Score	*p*-Value
	Md	Min	Max	Md	Min	Max			
Subjective sleep quality	1	0	3	1	0	3	5695.00	−0.54	0.591
Latency to sleep	1	0	3	2	0	3	5552.50	−0.77	0.441
Sleep duration	0	0	3	0	0	3	5539.00	−0.48	0.630
Habitual sleep efficiency	0	0	3	0	0	3	5650.50	−0.16	0.873
Sleep disturbances	1	1	3	1	0	3	5481.00	−1.00	0.317
Use of medication	0	0	3	0	0	3	5874.50	−0.18	0.859
Daytime dysfunction	2	0	3	2	0	3	5871.50	−0.12	0.908
**Global sleep score**	**6**	**2**	**18**	**7**	**1**	**14**	**5521.00**	−**0.80**	**0.425**

**Table 3:** PSQI component and global scores in accordance with each case study. Global scores greater than 5 indicate severe sleep deficits.

Case	PSQI Component Scores							
	SSQ	SL	SDU	HSE	SDI	SM	DD	Global Score
1	1	2	0	3	1	0	1	**8**
2	1	0	1	1	1	0	1	**5**

Notes: SSQ = subjective sleep quality; SL = sleep latency; SDU = sleep duration; HSE = habitual sleep efficiency; SDI = sleep disturbances; SM = use of sleeping medication; DD = daytime dysfunction.

**Table 4:** Epoch count (*n*) and percentage (%) of sleep–wake concordance based on when the human and dog is sleeping and/or awake.

Case Study	HS/DW		HW/DS		HS/DS		HW/DW	
	*n*	%	*n*	%	*n*	%	*n*	%
1	958	12.1	166	2.1	6656	83.4	107	1.4
2	985	14.9	344	5.2	4996	75.7	271	4.1

Notes: HS = human sleeping; HW = human awake; DW = dog awake; DS = dog sleeping.

## Data Availability

Survey data for Study 1 are available on request. Actigraphy data for Study 2 are available in Supplementary Materials.

## Supplementary Materials

The following are available online at https://www.mdpi.com/2624-5175/3/1/1/s1. Demographics survey and case study actigraphy reports.

## Abbreviations

PSQI	Pittsburgh Sleep Quality Index
