# Supplementary material for: Co-Sleeping between Adolescents and Their Pets May Not Impact Sleep Quality
Source: Clocks Sleep. 2021 Jan 4;3(1):1–11. doi: 10.3390/clockssleep3010001 (PMC7838871; doi:10.3390/clockssleep3010001)
Supplement: Supplementary file 1 [file clockssleep-03-00001-s001.zip › clockssleep-1004867 suppl/SUPP MAT Demographics survey.pdf]

DEMOGRAPHICS:

1. How old are you? (*please write number*) ______

2. What is your gender?
    - ➢ Female
    - ➢ Male
    - ➢ Non-binary/third gender
    - ➢ Prefer not to say

3. Do you have a pet?
    - ➢ Yes
    - ➢ No

If 'No' Skip to end of survey

4. What type of pet(s) do you have? Please select all that apply
    - ☐ Cat
    - ☐ Dog
    - ☐ Other type of pet (please write species) _______

(if cat owner)

5. How many cats do you have?
    - ➢ One
    - ➢ Two
    - ➢ Three +

6. How many of your cats sleep in your room at least some of the time?
    - ➢ None
    - ➢ One
    - ➢ Two
    - ➢ Three +

(if dog owner)

7. How many dogs do you have?
    - ➢ One
    - ➢ Two
    - ➢ Three +

(if one dog)

8. What is the size of your dog?
    - ➢ Small
    - ➢ Medium
    - ➢ Large

(if two or more dogs)

9. What is the size of your dog? Please select the dog that is most likely to sleep in your room at night. If none of your dogs sleep with you, select the oldest dog.
- ➢ Small
- ➢ Medium
- ➢ Large

10. How many of your dogs sleep in your room at least some of the time?
- ➢ None
- ➢ One
- ➢ Two
- ➢ Three +

(for all respondents)

11. How many nights does at least one of your pets sleep in the same **room** as you during a typical week?
- ➢ None
- ➢ One
- ➢ Two
- ➢ Three
- ➢ Four
- ➢ Five
- ➢ Six
- ➢ Seven

(if one or more)

12. Approximately how long does at least one of your pets sleep in the same room with you during a typical night?
- ➢ Less than 1 hour
- ➢ 1-3 hours
- ➢ 4-7 hours
- ➢ 8-10 hours
- ➢ More than 10 hours

13. How many nights does your pet/s sleep on the **bed** with you during a typical week?
- ➢ None
- ➢ One
- ➢ Two
- ➢ Three
- ➢ Four
- ➢ Five
- ➢ Six
- ➢ Seven

(if one or more)

14. Approximately how long does your pet/s sleep on your bed with you during a typical night?
- ➢ Less than 1 hour
- ➢ 1-3 hours
- ➢ 4-7 hours
- ➢ 8-10 hours
- ➢ More than 10 hours

15. What is your bed size?

- Single
- King single
- Double
- Queen
- King

16. How often do you sleep in your <u>own</u> bed?

- Once per month or less
- A few times per month
- Once or twice a week
- Three to four times per week
- Five to six times a week
- Every night

11. How many people, including yourself, live in your household?

- 1
- 2-3
- 4-5
- 6 +
